# Self-reported Symptoms of Depression Among Chinese Rural-to-Urban Migrants and Left-Behind Family Members

**DOI:** 10.1001/jamanetworkopen.2019.3355

**Published:** 2019-05-03

**Authors:** Zlatko Nikoloski, Anwen Zhang, Gareth Hopkin, Elias Mossialos

**Affiliations:** 1 Department of Health Policy, London School of Economics and Political Science, London, United Kingdom; 2 Institute of Health Economics, Edmonton, Alberta, Canada

## Abstract

**Importance:**

There were an estimated 247 million rural-to-urban migrant workers in China in 2016, yet at a national level, there is scant evidence on the association of migration with mental health among migrants and their left-behind family members.

**Objective:**

To examine the association of rural-to-urban migration with symptoms of depression among migrants and left-behind family members aged 45 years and older.

**Design, Setting, and Participants:**

Using representative cross-sectional data of 14 332 middle-aged and older adults from the 2015 China Health and Retirement Longitudinal Survey, regression analyses were conducted to examine the association of depressive symptoms with rural-to-urban migration status in urban areas and the association of depressive symptoms with left-behind status in rural areas. The statistical analysis was performed from January to August 2018.

**Exposures:**

Migration status (defined as having a rural *hukou* [household registration record]) in urban areas and left-behind status (defined as having a spouse or child living in another area) in rural areas.

**Main Outcomes and Measures:**

Depressive symptoms measured on the 10-item Center for Epidemiological Studies Depression (CES-D-10) scale.

**Results:**

A total of 14 332 middle-aged and elderly participants (mean [SD] age, 59.84 [9.51] years; 7394 [51.6%] women) were included, of whom 4404 (30.7%) lived in urban areas and 9928 (69.3%) lived in rural areas. In urban areas, 1607 participants (36.2%) were rural-to-urban migrants, and the remaining 2797 participants (72.8%) were local residents. In rural areas, 3405 participants (34.3%) were left-behind family members, and the remaining 6523 participants (65.7%) were not. Compared with urban residents, rural-to-urban migrants had higher CES-D-10 scores after adjustment for covariates (β = 0.74; 95% CI, 0.08-1.40; *P* = .03; standard errors clustered at the household level henceforth). Compared with intact-family rural residents, left-behind spouses had higher CES-D-10 scores after adjustment for covariates (β = 0.54; 95% CI, 0.05-1.03; *P* = .03).

**Conclusions and Relevance:**

Rural-to-urban migration in China was associated with poor mental health for migrants and their left-behind spouses. Short-term policies, such as building community social facilities, may prove effective, but long-term solutions should address issues related to economic and social exclusions and the lack of a social security system in rural China.

## Key Points

**Question:**

What is the association of rural-to-urban migration in China with symptoms of depression among migrants and their left-behind family members?

**Findings:**

In this study of cross-sectional data from the 2015 China Health and Retirement Longitudinal Survey including 14 332 middle-aged and older adults, rural-to-urban migration was associated with higher depressive symptoms, and a similar association was found among left-behind spouses.

**Meaning:**

Mental health policy in China should address the increased risk of symptoms of depression among migrants and their left-behind family members.

## Teaser

This cross-sectional study analyzes the association of rural-to-urban migration in China with mental health among migrants and their left-behind family members.

## Introduction

Rural-to-urban migration in China has been a major driving force behind its economic growth in the past few decades. Beyond economic implications, there has been an increasing interest from research and policy communities to understand the association of rural-to-urban migration with various well-being outcomes in China. The 2018 World Happiness Report^1^ suggests that rural-to-urban migrants report lower happiness levels despite their higher incomes. The associations of migration could go beyond happiness, well-being, and life satisfaction: migration could also be associated with worse health outcomes, including mental health outcomes.

Despite increased interest, the literature on the association of rural-to-urban migration with mental health and the channels through which rural-to-urban migration and mental health are associated is limited, particularly for older adults. To our knowledge, most of the literature has focused on the migration and mental health nexus of younger migrants.^2^ In addition, the literature offers limited evidence on the channels through which migration and mental health are associated. To date, most studies in the literature focus on the *hukou* system, the Chinese household registration system, which is primarily based on place of birth and hence divided into 2 broad categories: urban and rural. *Hukou* affiliation is linked to the provisioning of social services (eg, schooling, types of jobs and employee benefits, housing opportunities).^3^ This implies that rural-to-urban migrants are generally excluded from social welfare services,^4,5,6^ with a potential impact on mental health, particularly for women and now-older migrants who moved during the earlier, more restrictive *hukou* environment.^7,8,9^ For older rural-to-urban migrants who faced a more restrictive system, this may be associated with poorer mental health compared with their younger counterparts. A 2014 study^2^ of a single city in China supports this notion, but nationally representative samples have not been explored, to our knowledge.

The large wave of rural-to-urban migration has also created a group of left-behind parents, spouses, and older adults. This issue has been explored in other Asian countries, but, to our knowledge, most of the studies in the Chinese context have been small and nonrepresentative.^10,11^ The existing studies in China suggest that older left-behind or empty-nest parents have higher levels of depressive symptoms. Higher depressive symptoms may be attributed to lowered social capital, worse economic status, and increased loneliness.^12,13,14^ More evidence from a nationally representative survey is needed to examine the mental health status of left-behind family members and the potential mitigation mechanisms, such as remittances or close emotional ties.^15,16^

Additionally, further evidence is needed to distill policies relevant for strengthening the mental health of elderly rural-to-urban migrants. For example, more emphasis may be needed on community building and promotion of social support networks.^17^ Studies have shown that increased social participation is associated with higher subjective well-being and can mediate the association of resources with well-being for retired people.^18,19,20^ It is important to examine whether this is also the case for elderly migrant groups.

This study had 3 objectives. The first was to examine the association of migration status with depressive symptoms in urban areas. The second was to examine the association of left-behind status with depressive symptoms in rural areas. The third was to explore factors that could contribute to or mitigate the disparities in depressive symptoms associated with rural-to-urban migration.

## Methods

### Data Source and Study Sample

Our sample came from the household questionnaire administered in the 2015 China Health and Retirement Longitudinal Study (CHARLS).^21^ The CHARLS is a nationally representative survey of individuals in China aged 45 years and older, providing rich data to serve the needs of scientific research on older adults. The national baseline survey for the CHARLS was conducted between June 2011 and March 2012 and included 17 708 individual participants. Samples were chosen through multistage probability sampling. In the first stage, 150 county-level units were randomly chosen with a probability-proportional-to-size sampling technique from a sampling frame containing all county-level units except Tibet. The sample was stratified by region, within region by urban districts or rural counties, and per capita on gross domestic product. The final sample included 150 counties in 28 provinces. In the second stage, the survey used villages in rural areas and communities in urban areas as primary sampling units. Three primary sampling units were selected within each county-level unit using probability-proportional-to-size sampling. Finally, 80 households were randomly sampled in each primary sampling unit, from which those with an age-eligible member (≥45 years) were approached by survey collectors for participation. Written informed consent and individual information were collected through computer-assisted personal interviewing. Since CHARLS Wave1 was completed in 2012, 2 follow-up surveys have been conducted to date, one in 2013 (CHARLS Wave2) and another in 2015 (CHARLS Wave3), which tracked previous respondents and included a small share of new respondents. We used the most recent data from the 2015 wave for our study. The 2015 wave included 20 284 participants in total, from which we selected 14 332 after excluding 615 age-ineligible participants, 2484 participants in semiurban areas, and 2853 participants with missing values. We split the data into an urban subsample for the analysis of rural-to-urban migrants vs urban residents and a rural subsample for the analysis of left-behind rural family members vs intact-family rural residents. Ethical approval for primary data collection was obtained from the Ethical Review Committee at Peking University in 2011. Our research involved secondary analysis of deidentified established data sets and was not subject to ethical approval or informed consent according to London School of Economics and Political Science^22^ research ethics policy and procedures. Results were reported following the Strengthening the Reporting of Observational Studies in Epidemiology (STROBE) reporting guideline for cross-sectional studies.

### Key Variable Definitions

#### Depressive Symptoms

Depressive symptoms were measured with the 10-item Center for Epidemiologic Studies Depression (CES-D-10) scale.^23,24^ Respondents were asked to rate how often they experienced depressive symptoms in the last week, with 0 indicating rarely, 1 indicating some days (1-2 days), 2 indicating occasionally (3-4 days), and 4 indicating most of the time (5-7 days). The responses were summed to produce a score from 0 to 30. A binary measure for scoring 10 or greater was used as a robustness check.^24^

#### Migrants

We based our analysis of migrants on the urban subsample. Urban and rural area definitions were provided in the CHARLS according to the guidelines by the China National Bureau of Statistics.^25^ Out of the concern that urban areas by this definition include the outskirts of city and town areas that were in fact rural in nature,^26^ we excluded the outskirt areas and restricted the urban sample to the central areas of cities and towns. Migration status was defined by respondents’ current place of residence and their current *hukou* status. Individuals were categorized as rural-to-urban migrants if they lived in an urban area and currently had rural *hukou*. Individuals were categorized as urban residents if they lived in an urban area and had nonrural *hukou*. In further analysis, we divided rural-to-urban migrants by sex.

#### Left-Behind Family Members

We based our analysis of left-behind family members on the rural subsample. Left-behind spouses were people living in a rural area who did not live with their spouse. Left-behind parents were people who had no children living nearby (in the same household or community) and at least 1 child who was not living in the same community. A left-behind family member was defined as being either a left-behind spouse or left-behind parent. Other rural residents were categorized as intact-family rural residents. Left-behind spouses and left-behind parents were not necessarily mutually exclusive.

#### Covariates

Covariates were selected to control for potential confounding related to socioeconomic status and physical health. Socioeconomic variables included sex, age, marital status (currently married or not), education levels (4 categories: primary school and below, middle school, high school and above, and not reported), and a continuous variable of logarithm of per-capita household expenditure (coded as 0 if not reported). Physical health indicators included a binary variable of having fallen down in the last 2 years (to proxy for physical injury), a binary variable of experiencing any physical function difficulty in 10 daily tasks (eg, walking 1 kilometer), and a binary indicator of having been diagnosed as having 1 or more of 13 listed long-term conditions (eg, diabetes). A binary variable was also included to indicate if physical function difficulty information was not reported.

#### Additional Variables

We further considered variables about economic and social exclusion in parts of the analysis. For rural-to-urban migrants, these included a binary variable indicating owning their current residence, a binary variable indicating having any savings, a binary variable indicating engaging at least weekly in 1 or more of 8 listed social activities (eg, visiting friends), a community social facilities index (range, 0-1) indicating the availability of 1 or more of 14 listed facilities in the village or community (eg, an elderly center), and years since migration. For left-behind family members, these included 2 variables of receiving support: a binary variable indicating having received financial support (in cash or in kind) from children, and a binary variable indicating seeing children at least once per year.

### Statistical Analysis

#### Descriptive Statistics

We started by presenting descriptive statistics. First, we showed regional variations in depressive symptoms among provinces by plotting the provincial CES-D-10 mean score on a map. We divided the provincial mean CES-D-10 scores into quartiles, then visualized the data by displaying the results on a thematic map. Second, we reported unconditional subgroup means in urban and rural areas separately. Specifically, for the urban subsample, we compared the mean CES-D-10 scores and the proportions of CES-D-10 scores of 10 or greater of rural-to-urban migrants with urban residents, and for the rural subsample, we compared mean CES-D-10 scores of left-behind with intact-family rural residents. We used *t* tests to test the differences.

#### Association of Migration or Left-Behind Status With Depressive Symptoms

Least squares regression analyses were conducted to assess the association of migration or left-behind status with depressive symptoms. To allow for error terms to be associated within the household, we clustered standard errors at the household level to avoid overstating estimation precision. Survey sampling weights were applied in all regressions. Analyses were performed using Stata statistical software version 14.2 (StataCorp).

Next, we explored what factors might be associated with the potential disparities in depressive symptoms by migration status. We regressed CES-D-10 scores based on rural-to-urban migrant status, while also controlling for a range of socioeconomic and health status covariates. In recognition of regional disparities of economic and social development, we further controlled for province fixed effects. We further examined heterogeneity by replacing the rural-to-urban migrant variable with 2 variables, one indicating a male migrant and the other indicating a female migrant.

To assess potential migration-associated depressive symptoms pathways, we focused on the economic and social exclusion of migrants by regressing an economic or social exclusion indicator based on migration status. The indicators included whether the respondent owned the property of current residence, whether the respondent had any individual savings, and a social activity index. We next considered what factors might reduce the risk of depressive symptoms for migrants. Focusing on the migrant subsample, we analyzed if certain factors (ie, presence of community social facilities and years of residence in the migration destination) were associated with lower depressive symptoms among migrants.

Last, we examined whether migration was associated with increased depressive symptoms for left-behind family members. In the rural sample, we regressed CES-D-10 scores based on left-behind status and covariates. In an alternative specification, we replaced the left-behind status variable with left-behind spouse status or left-behind parent status. In the left-behind sample, we regressed CES-D-10 based on 2 variables indicating financial and emotional support and covariates to understand the association of depressive symptoms with familial support for left-behind family members.

*P* values were 2-tailed. Statistical significance was set at less than .05.

## Results

A total of 14 332 middle-aged and elderly participants (mean [SD] age, 59.84 [9.51] years; 7394 [51.6%] women) were included, of whom 4404 (30.7%) lived in urban areas and 9928 (69.3%) lived in rural areas. In urban areas, 1607 participants (36.2%) were rural-to-urban migrants, and the remaining 2797 participants (72.8%) were local residents. In rural areas, 3405 participants (34.3%) were left-behind family members, and the remaining 6523 participants (65.7%) were not. Descriptive statistics for urban, rural, and all participants are shown in Table 1.

**Table 1.** Descriptive Statistics for All Variables Among Urban, Rural, and All Participants^a^

Characteristic	Mean (SD)		
	Urban (n = 4404)	Rural (n = 9928)	Total (N = 14 332)
Depressive symptoms^b^			
CES-D-10 score, points	6.88 (6.05)	8.79 (6.67)	8.20 (6.54)
CES-D-10 score ≥10	0.26 (0.44)	0.38 (0.49)	0.35 (0.48)
Migration status			
Migrant	0.36 (0.48)	NA	NA
Female migrant	0.20 (0.40)	NA	NA
Male migrant	0.16 (0.37)	NA	NA
Economic and social exclusion			
Own current residence	0.85 (0.35)	NA	NA
Have any savings	0.42 (0.49)	NA	NA
Weekly social activities	0.43 (0.50)	NA	NA
Community facilities index	0.48 (0.25)	NA	NA
Time living in current community, y	32.76 (22.51)	NA	NA
Sociodemographic characteristic			
Women	0.53 (0.50)	0.51 (0.50)	0.52 (0.50)
Age, y	60.19 (9.74)	59.68 (9.40)	59.84 (9.51)
Married	0.87 (0.33)	0.87 (0.34)	0.87 (0.34)
Education			
Primary school and below	0.39 (0.49)	0.67 (0.47)	0.59 (0.49)
Middle school	0.24 (0.43)	0.17 (0.37)	0.19 (0.39)
High school and above	0.22 (0.41)	0.05 (0.23)	0.10 (0.30)
Not reported	0.16 (0.37)	0.11 (0.31)	0.12 (0.33)
Household expenditure			
Logarithm of household expenditure, ¥	8.59 (3.28)	8.13 (3.19)	8.28 (3.23)
Not reported	0.12 (0.33)	0.12 (0.33)	0.12 (0.33)
Physical health			
Recently fell down	0.15 (0.35)	0.18 (0.39)	0.17 (0.38)
Physical function difficulty	0.39 (0.49)	0.46 (0.50)	0.44 (0.50)
Long-term disease	0.72 (0.45)	0.74 (0.44)	0.73 (0.44)
Not reported	0.03 (0.16)	0.02 (0.15)	0.02 (0.15)
Left-behind relationship			
Family member	NA	0.34 (0.48)	NA
Spouse	NA	0.06 (0.25)	NA
Parent	NA	0.31 (0.46)	NA
Support received from children			
Financial	NA	0.61 (0.49)	NA
Emotional^c^	NA	0.72 (0.45)	NA

Abbreviations: CES-D-10, 10-item Center for Epidemiological Studies Depression scale; NA, not applicable.

^a^ Variables without units are binary variables, and the means reported refer to the proportions of taking value 1.

^b^ Possible range of CES-D-10 scores is 0 to 30, with higher scores indicating more severe depressive symptoms.

^c^ Defined as seeing children once per year.

Graphical evidence suggested there were wide variations in depressive symptoms among provinces in China, with the mean (SD) provincial CES-D-10 score ranging from 4.41 (3.86) points to 12.32 (6.96) points. There was a geographical trend that depressive symptoms increased from east to west (Figure). This roughly corresponded with decreasing economic development levels from east to west.^27^

**Figure.**
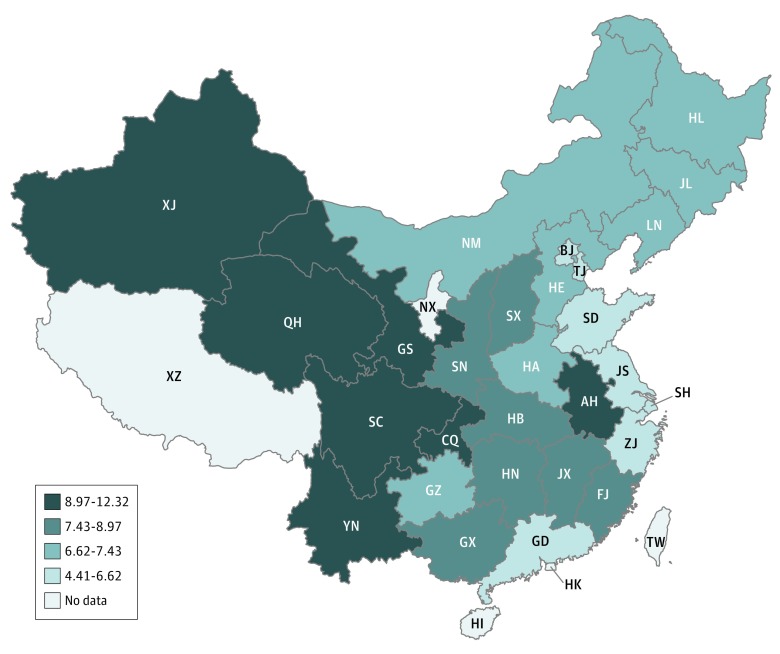
Mean 10-Item Center for Epidemiological Studies Depression Scale (CES-D-10) Scores in 28 Provinces in China, 2015 Provincial-level mean CES-D-10 scores are estimated from individual-level data with survey sampling weights. Possible range of CES-D-10 scores is 0 to 30, with higher scores indicating more severe depressive symptoms. Twenty-eight provincial units with valid data are colored and divided into 4 categories by quartiles. AH indicates Anhui; BJ, Beijing; CQ, Chongqing; FJ, Fujian; GD, Guangdong; GS, Gansu; GX, Guangxi; GZ, Guizhou; HA, Henan; HB, Hubei; HE, Hebei; HI, Hainan; HK, Hong Kong; HL, Heilongjiang; HN, Hunan; JL, Jilin; JS, Jiangsu; JX, Jiangxi; LN, Liaoning; NM, Inner Mongolia; NX, Ningxia; QH, Qinghai; SC, Sichuan; SD, Shandong; SH, Shanghai; SN, Shaanxi; SX, Shanxi; TJ, Tianjin; TW, Taiwan; XJ, Xinjiang; XZ, Tibet; YN, Yunnan; ZJ, Zhejiang.

Rural-to-urban migrants reported higher mean CES-D-10 scores than urban residents among both men and women. Without adjusting for covariates, male migrants’ CES-D-10 scores were greater than male urban residents’ scores (mean [SD], 6.17 [5.75] points vs 5.50 [4.95] points; β = 0.67; 95% CI, −0.42 to 1.77; *P* = .23), which is 10% of 1 SD of the whole distribution (6.54 points). The difference between CES-D-10 scores of female migrants and female urban residents was higher (mean [SD], 8.67 [7.02] points vs 6.88 [5.69] points; β = 1.80; 95% CI, 0.39-3.20; *P* = .01), or 27% of 1 SD. Male migrants had a probability (SD) of 0.22 (0.42) of reporting high depressive symptoms (CES-D-10 score ≥10) compared with their urban counterparts’ probability (SD) of 0.17 (0.37). Among female migrants, the probability (SD) of reporting high depressive symptoms was 0.40 (0.49) compared with their urban counterparts’ probability (SD) of 0.26 (0.44).

Table 2 shows the results of our analysis of the difference in depressive symptoms between rural-to-urban migrants and urban residents and the associated factors. Compared with urban residents, rural-to-urban migrants had higher CES-D-10 scores after adjustment for covariates (β = 0.74; 95% CI, 0.08-1.40; *P* = .03). Compared with intact-family rural residents, left-behind spouses had higher CES-D-10 scores after adjustment for covariates (β = 0.54; 95% CI, 0.05-1.03; *P* = .03). Being a woman and being in poor physical health (indicated by having recently fallen down, physical functional difficulty, or long-term disease) were associated with reporting higher depressive symptoms. Older age, being married, having higher education levels, and having higher living standards (indicated by household expenditure) were associated with reporting lower depressive symptoms. Estimated coefficients for covariates are reported in Table 1. Compared with female urban residents and conditional on covariates and provincial fixed effects, female migrants had higher CES-D-10 scores (β = 1.06; 95% CI, 0.23-1.89; *P* = .01). Compared with male urban residents and conditional on covariates and provincial fixed effects, male migrants also had higher CES-D-10 scores (β = 0.40; 95% CI, −0.37 to 1.18; *P* = .31).

**Table 2.** Scores on CES-D-10 for Rural-to-Urban Migrants Compared With Urban Residents, Adjusted by Sociodemographic Covariates^a^

Variable	CES-D-10 Score, β (95% CI)	
	Overall	By Sex
Migrant	0.74 (0.08 to 1.40)^b^	NA
Female migrant	NA	1.06 (0.23 to 1.89)^b^
Male migrant	NA	0.40 (−0.37 to 1.18)
Women	0.83 (0.40 to 1.25)^c^	0.62 (0.10 to 1.14)^b^
Age	−0.06 (−0.10 to −0.03)^c^	−0.06 (−0.10 to −0.03)^c^
Married	−1.21 (−1.98 to −0.44)^c^	−1.23 (−1.10 to −0.46)^c^
Education level		
Primary school and below	[Reference]	[Reference]
Middle school	−0.83 (−1.46 to −0.20)^c^	−0.80 (−1.43 to −0.17)^b^
High school and above	−0.82 (−1.44 to −0.20)^c^	−0.81 (−1.44 to −0.19)^b^
Not reported	0.18 (−0.67 to 1.04)	0.18 (−0.67 to 1.04)
Household expenditure		
Logarithm of expenditure	−0.37 (−0.74 to 0.001)^d^	−0.37 (−0.74 to 0)^d^
Not reported	−3.33 (−7.03 to 0.37)^d^	−3.35 (−7.04 to 0.35)^d^
Physical health		
Recently fell down	2.14 (1.39 to 2.88)^c^	2.14 (1.39 to 2.89)^c^
Physical function difficulty	3.12 (2.55 to 3.68)^c^	3.12 (2.55 to 3.68)^c^
Long-term disease	1.19 (0.58 to 1.79)^c^	1.16 (0.55 to 1.77)^c^
Not reported	−2.14 (−3.30 to −0.98)^c^	−2.13 (−3.28 to −0.98)^c^

Abbreviations: CES-D-10, 10-item Center for Epidemiological Studies Depression scale; NA, not applicable.

^a^ A constant term and a set of binary indicators for provinces were also included in the covariates but not reported here. Standard errors were clustered at the household level. Sampling weights were applied.

^b^ *P* < .05.

^c^ *P* < .01.

^d^ *P* < .10.

Table 3 shows regression results in which a number of economic and social exclusion variables were examined. Based on the urban sample, migrant status was associated with 13.4% (95% CI, −0.19 to −0.07; *P* < .001) lower probability of owning current residence, 10.3% (95% CI, −0.17 to −0.04; *P* < .001) lower probability of having any savings, and 8.6% (95% CI, −0.14 to −0.03; *P* < .001) lower probability of engaging in weekly social activities. Statistical significance remained for these 3 multiple tests after applying Bonferroni correction. Compared with migrants who did not live in a community with community facilities, migrants living in a community with community facilities had lower CES-D-10 scores (β = −3.02; 95% CI, −4.70 to −1.34; *P* < .001). More years since migration was not associated with CES-D-10 (β = 0; 95% CI, −0.02 to 0.01; *P* = .68).

**Table 3.** Socioeconomic Exclusion Indicators of Rural-to-Urban Migrants Compared With Urban Residents, Adjusted by Socioeconomic Covariates^a^

Variable	Socioeconomic Exclusion Indicator, β (95% CI)			CES-D-10 Score, β (95% CI)
	Own Current Residence (n = 4299)	Have Any Saving (n = 4266)	Weekly Social Activity Index (n = 4259)	
Migrant	−0.13 (−0.19 to −0.07)^b^	−0.10 (−0.17 to −0.04)^b^	−0.09 (−0.14 to −0.03)^b^	NA
Community social facilities index	NA	NA	NA	−3.02 (−4.70 to −1.34)^b^
Years since migration	NA	NA	NA	−0.004 (−0.02 to 0.01)
Female	−0.002 (−0.03 to 0.02)	−0.05 (−0.09 to −0.004)^c^	−0.01 (−0.05 to 0.04)	1.28 (0.53 to 2.04)^b^
Age	−0.002 (−0.004 to 0.001)	0.002 (0 to 0.01)	0 (−0.003 to 0.002)	−0.02 (−0.07 to 0.03)
Married	0.08 (0.02 to 0.14)^b^	0.07 (0.01 to 0.13)^c^	−0.02 (−0.09 to 0.04)	−1.91 (−3.36 to −0.46)^b^
Education level				
Primary school and below	[Reference]	[Reference]	[Reference]	[Reference]
Middle school	−0.003 (−0.06 to 0.06)	0.04 (−0.02 to 0.11)	0.04 (−0.02 to 0.10)	−0.99 (−2.13 to 0.15)^d^
High school and above	−0.01 (−0.07 to 0.05)	0.11 (0.05 to 0.17)^b^	0.13 (0.06 to 0.19)^b^	−0.45 (−1.76 to 0.86)
Not reported	−0.11 (−0.20 to −0.02)^c^	0.06 (−0.04 to 0.16)	0.10 (0.01 to 0.19)^c^	1.23 (−0.09 to 2.55)
Household expenditure				
Logarithm of expenditure	0.01 (−0.03 to 0.04)	0.06 (0.03 to 0.09)^b^	0.042 (0.01 to 0.07)^b^	0.08 (−0.51 to 0.67)^d^
Not reported	0.06 (−0.27 to 0.39)	0.53 (0.23 to 0.83)^b^	0.41 (0.11 to 0.70)^b^	0.72 (−4.78 to 6.22)
Physical health				
Recently fell down	−0.004 (−0.06 to 0.05)	−0.01 (−0.07 to 0.05)	0.01 (−0.05 to 0.08)	2.31 (1.14 to 3.47)^b^
Physical function difficulty	0.01 (−0.04 to 0.05)	−0.05 (−0.10 to −0.01)^c^	−0.09 (−0.14 to −0.05)^b^	3.55 (2.58 to 4.53)^b^
Long-term disease	−0.04 (−0.09 to 0.01)	0.01 (−0.05 to 0.06)	0.02 (−0.04 to 0.09)	1.32 (0.42 to 2.21)^b^
Not reported	−0.004 (−0.15 to 0.14)	0.16 (−0.01 to 0.34)^d^	0.13 (−0.04 to 0.30)	−0.49 (−3.19 to 2.21)

Abbreviations: CES-D-10, 10-item Center for Epidemiological Studies Depression scale; NA, not applicable.

^a^ A constant term and a set of binary indicators for provinces were included in the covariates but not reported here. Standard errors were clustered at the household level. Sampling weights were applied.

^b^ *P* < .01.

^c^ *P* < .05.

^d^ *P* < .10.

Finally, we explored the mental health associations among the family members left behind because of spouse or adult child migration, as reported in Table 4. Compared with those not left behind, left-behind spouses had significantly higher CES-D-10 scores (β = 0.54; 95% CI, 0.05-1.03; *P* = .03), as did left-behind parents (β = 0.05; 95% CI, −0.25 to 0.35; *P* = .74), although the difference was not significant. Among the left-behind sample, compared with people who did not receive financial support from family, people who received financial support from family had lower CES-D-10 scores (β = −0.48; 95% CI, −1.05 to 0.09; *P* = .10), and compared with not seeing children at least once per year, left-behind people who did see their children at least once per year had lower CES-D-10 scores (β = −0.54; 95% CI, −1.06 to −0.03; *P* = .04).

**Table 4.** Scores on CES-D-10 for Left-Behind Family Members Compared With Intact-Family Rural Residents, Adjusted for Covariates^a^

Explanatory Variable	CES-D-10, β (95% CI)		
	Overall	By Relationship	Among Left-Behind Family Members by Receipt of Family Support
Left-behind family member	0.15 (−0.14 to 0.44)	NA	NA
Left-behind spouse	NA	0.54 (0.05 to 1.03)^b^	NA
Left-behind parent	NA	0.05 (−0.25 to 0.35)	NA
Receive financial support from children	NA	NA	−0.48 (−1.05 to 0.09)^c^
See children ≥1 time per year	NA	NA	−0.54 (−1.06 to −0.03)^b^
Female	1.34 (1.10 to 1.58)^d^	1.34 (1.09 to 1.58)^d^	1.22 (0.82 to 1.62)^d^
Age	−0.04 (−0.06 to −0.02)	−0.04 (−0.05 to −0.02)^d^	−0.02 (−0.05 to 0.01)
Married	−1.79 (−2.24 to −1.34)^d^	−1.81 (−2.26 to −1.36)^d^	−2.03 (−2.96 to −1.10)^d^
Education level			
Primary school and below	[Reference]	[Reference]	[Reference]
Middle school	−0.65 (−1.00 to −0.30)^d^	−0.65 (−1.00 to −0.30)^d^	−0.88 (−1.41 to −0.36)^d^
High school and above	−1.30 (−1.83 to −0.78)^d^	−1.31 (−1.83 to −0.78)^d^	−1.45 (−2.28 to −0.63^d^
Not reported	0.07 (−0.40 to 0.53)	0.05 (−0.41 to 0.51)	−0.22 (−0.98 to 0.54)
Household expenditure			
Logarithm of expenditure	−0.22 (−0.38 to −0.06)^d^	−0.21 (−0.38 to −0.05)^b^	0.08 (−0.19 to 0.36)
Not reported	−2.01 (−3.59 to −0.44)^b^	−1.96 (−3.53 to −0.38)^b^	1.06 (−1.64 to 3.75)
Physical health			
Recently fell down	2.23 (1.86 to 2.60)^d^	2.24 (1.87 to 2.60)^d^	2.29 (1.68 to 2.90)^d^
Physical function difficulty	3.08 (2.79 to 3.38)^d^	3.08 (2.79 to 3.38)^d^	3.09 (2.59 to 3.59)^d^
Long-term disease	1.42 (1.12 to 1.72)^d^	1.42 (1.12 to 1.72)^d^	1.17 (0.68 to 1.66)^d^
Not reported	−1.59 (−2.31 to −0.87)^d^	−1.58 (−2.30 to −0.86)^d^	−1.35 (−2.54 to −0.16)^b^

Abbreviations: CES-D-10, 10-item Center for Epidemiological Studies Depression scale; NA, not applicable.

^a^ A constant term and a set of binary indicators for provinces were included in the covariates but not reported here. Standard errors were clustered at the household level. Sampling weights were applied.

^b^ *P* < .05.

^c^ *P* < .10.

^d^ *P* < .01.

## Discussion

Based on our analysis of data from the 2015 CHARLS survey, we report a number of important findings. Our analysis found that rural-to-urban migration was associated with higher depressive symptoms in middle and old age. This association was particularly strong among female migrants. We also found that migration was positively associated with economic deprivation (proxied by house ownership and savings) and social exclusion (proxied by social activities index), suggesting these factors were likely channels underlying the association of migration with depressive symptoms. We also found that community building (as proxied by community social facility index) could mitigate the negative association of migration with depressive symptoms, in that migrants living in communities with more social facilities had lower depressive symptoms than migrants living in communities with fewer social facilities. Additionally, we found that being a left-behind spouse was associated with higher depressive symptoms and that among left-behind parents, receiving more familial support from children financially or emotionally was associated with lower depressive symptoms than receiving less familial support.

Previous analyses have argued that female migrants have worse mental health status because of the nature of the *hukou* system and the marginalization of women within the system. Existing research argues that migrant women have multiple marginalized identities and thus are more likely to be exposed to social and economic inequality compared with their male counterparts.^8,9,28^ Although we were not able to show causal effects, our findings support the notion that female migrants may be at higher risk of depression than their male counterparts.

We explored the roles of economic and social exclusions in understanding the association of migration with depressive symptoms. These 2 mechanisms are particularly relevant to rural-to-urban migrants in China where the *hukou* system acts as an institutional barrier to integration.^29^ Previous studies have explored the urban economic and social exclusion of rural-to-urban migrants and the ways in which they are excluded.^4,5,6^ One study that used subjective measures of socioeconomic status showed that migrants had higher likelihood of economic isolation,^30^ while another found migration led to divided social identities and social stigma.^31^ Our results support the literature that suggests that economic and social deprivation could translate into poor mental health. From a policy perspective, it is important to understand what resources and facilities might mitigate the negative association of migration with mental health. We contributed to this question by showing that the availability of community social facilities could be associated with better mental health outcomes among migrants.^32,33^

While we did not find evidence that left-behind family members were associated with worse mental health status overall, we did observe that left-behind spouses were associated with higher depressive symptoms and left-behind parents were not. Among left-behind parents, our results suggest that familial support is important for lowering depressive symptoms. Because of the lack of a comprehensive pension system in rural China, family support is the primary source of support for elderly rural residents.^34^ Within this context, intergenerational support structures, such as financial support and regular visits from children, play an important role for the mental health of the rural left-behind elderly population.

### Strengths and Limitations

There are a few strengths of our work. First, this analysis is the first analytical effort on the mental health and migration nexus of middle-aged and elderly Chinese people relying on a well-established nationally representative data set, to our knowledge. Second, in performing our analysis, we also explored some of the factors that could be associated with lower mental health outcomes among elderly migrants, such as socioeconomic exclusion. Relevant to policy making, we also explored whether the availability of community social facilities could help limit the negative association of migration with mental health. Third, we emphasized that migration was not only associated with the mental health of the migrants themselves but also with the mental health of family members that were left behind. We further explored the association of familial support with the mental health of left-behind family members.

This study also had limitations. First, although we tried to control for as many confounding covariates and regional variations as possible, there were unobservable factors that might be associated with migration and mental health that we were not able to account for. Thus, our interpretation of the results was not causal. Although we did not establish causality, we did show a pattern of association of migration with mental health status. Second, our data only included individuals aged 45 years and older, so we were unable to examine how the mental health of left-behind children might be associated with the rural-to-urban migration of their parents.

## Conclusions

Mental health policy in China should address the issue that migrants and some of their left-behind family member may be at higher risk of poor mental health. Effectively addressing this issue requires more broad policy coordination to deal with economic and social exclusions associated with rural-to-urban migration. While this will be a long-term task, our findings regarding the association of community-based social facilities with mental health provides support to the notion that community-based social facilities could contribute to improving mental health of elderly migrants by facilitating opportunities for interaction with others and increasing feelings of self-determination. Community building projects should be continued, and access to community centers for rural-to-urban migrants should be facilitated. Additionally, familial support was associated with the mental health of left-behind family members. Although emotional support may be irreplaceable, the lack of a formal social security system in rural areas could be a factor in why left-behind rural residents rely on their children for financial support. Establishing a social security system in rural areas could be a path to economic security and mental well-being for rural residents.
